# A comparative study of simulated electric fields of transcranial magnetic stimulation targeting different cortical motor regions

**DOI:** 10.1002/bem.22523

**Published:** 2024-09-16

**Authors:** Jack Jiaqi Zhang, Bella Bingbing Zhang, Zhongfei Bai, Kenneth N. K. Fong

**Affiliations:** ^1^ Department of Rehabilitation Sciences The Hong Kong Polytechnic University Hung Hom Hong Kong SAR China; ^2^ Department of Neurology and Neurological Rehabilitation, Shanghai YangZhi Rehabilitation Hospital (Shanghai Sunshine Rehabilitation Centre), School of Medicine Tongji University Shanghai China

**Keywords:** electric fields, motor cortex, simulation, transcranial magnetic stimulation

## Abstract

This computational simulation study investigates the strength of transcranial magnetic stimulation (TMS)‐induced electric fields (EF) in primary motor cortex (M1) and secondary motor areas. Our results reveal high interindividual variability in the strength of TMS‐induced EF responses in secondary motor areas, relative to the stimulation threshold in M1. Notably, the activation of the supplementary motor area requires high‐intensity stimulation, which could be attributed to the greater scalp‐to‐cortex distance observed over this area. These findings emphasize the importance of individualized planning using computational simulation for optimizing neuromodulation strategies targeting the cortical motor system.

## Highlights

- There is an interindividual variability in the strength of transcranial magnetic stimulation‐induced electric fields in secondary motor areas.
- The strength of transcranial magnetic stimulation‐induced electric fields is related to the scalp‐to‐cortex distance observed over the stimulated area.
- The results emphasize the importance of individualized planning using computational simulation for optimizing transcranial brain stimulation targeting the cortical motor system.

Transcranial magnetic stimulation (TMS) has been extensively utilized as a neuroplasticity‐enhancing technique to facilitate functional recovery following neurological diseases (Somaa et al., 2022). Repetitive TMS protocols over the primary motor cortex (M1) have demonstrated a boosting effect on therapeutic gains from rehabilitation interventions (Zhang et al., 2022). Recently, secondary motor areas, such as the dorsal and ventral premotor cortex (PMd/PMv), supplementary motor area (SMA), and pre‐SMA have garnered attention. These targets are associated with high‐level cognitive‐motor functions, such as motor skills learning, motor (dis)inhibition, and motor planning, all of which are crucial for functional recovery after neurological diseases, such as stroke and Parkinson's disease (Somaa et al., 2022). Additionally, they play a significant role in reducing behavioral symptoms in patients with psychiatric disorders, such as obsessive‐compulsive disorder (Arumugham et al., 2018). The stimulation intensity applied to the secondary motor areas is usually determined based on the threshold of stimulation applied to the M1, that is, resting motor threshold (RMT). The stimulation intensity typically ranges from 90% to 110% RMT, depending on the specific research (Fiori et al., 2018; Matsunaga et al., 2005; Obeso et al., 2017; Wang et al., 2014). Nevertheless, the underlying justification for choosing this intensity for secondary motor areas has not been firmly established. Because the biological effect induced by TMS is associated with the electric field (EF) it generates (Saturnino et al., 2019), it would be feasible to determine the appropriate stimulation intensity by analyzing the simulated EF of TMS targeting different regions. Therefore, the current study aimed to establish the optimal stimulation intensity of four commonly selected regions within the cortical motor system (PMd, PMv, SMA, and pre‐SMA) by referencing the strength of EF induced by M1 stimulation.

**Figure 1:**
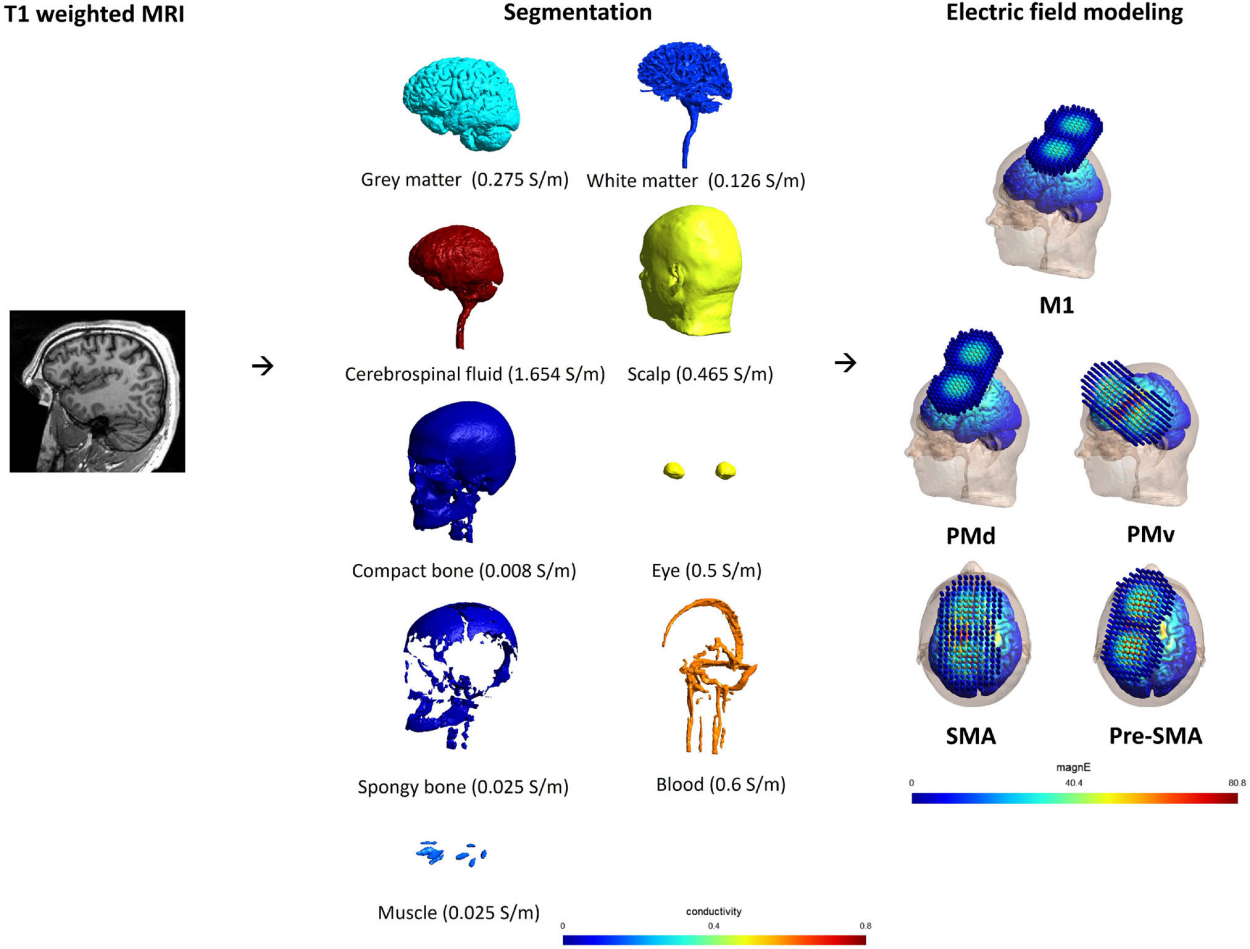
The standardized pipeline of electric field modeling. We first performed segmentation using the charm method using the individual T1‐weighted image. Then, we conducted transcranial magnetic stimulation‐induced electric field simulation over the different motor areas. SMA, supplementary motor area.

This simulation study was conducted based on a subsample of T1‐weighted MRI scans of young healthy adults (*n* = 30, 20–35 years old, 18 females) drawn from the OpenNeuro repository. The scanning parameters are described in the original publication (Zareba et al., 2022). The strength of TMS‐induced EF was simulated in anatomically realistic volume conductor head models by the finite element method (FEM) using SimNIBS version 4.0. The segmentation and meshing pipeline *charm* was used for head model reconstruction (Puonti et al., 2020) and the generated head models were visually inspected to avoid any segmentation errors (Saturnino et al., 2019). In SimNIBS version 4.0, the volume conductor model is constructed using tetrahedral elements. Our final meshes typically consist of approximately 700,000 nodes and 4,000,000 tetrahedra. The size of the tetrahedral elements is locally adapted such that elements that are far from tissue borders are larger and elements that are close to tissue borders are smaller. Concerning the mesh accuracy, the *charm* meshes have been previously validated (Puonti et al., 2016, 2020). Figure 1 depicts the pipeline of computational simulation.

A standard figure‐of‐eight shaped coil (MagVenture; Cool‐B65) was selected for the simulation. The TMS coil was positioned over four regions of interest (ROIs) with the following MNI coordinates: PMd (−30, −4, 58), PMv (−50, 5, 22), SMA (−2, −7, 55), and pre‐SMA (−3, 6, 53), as well as one reference target M1 with coordinates (−37, −21, 58) located in the left hemisphere (Mayka et al., 2006). The orientation of TMS coil placement was adjusted for each ROI following previous practice. Specifically, for M1 and PMd, the coil was angled 45° to the sagittal plane in a posterior‐to‐anterior direction (Wang et al., 2014). For the PMv, the coil was angled 45° to the sagittal plane in an anterior‐to‐posterior direction (Fiori et al., 2018). Finally, for the SMA and pre‐SMA, the coil was angled 90° to the sagittal plane in a lateral‐to‐medial direction (Matsunaga et al., 2005; Obeso et al., 2017). The coil orientations are almost perpendicular to the stimulated gyrus, which may lead to greater neuronal activation (Figure 1). The stimulation intensity during simulation modeling was set at 50% of the maximal machine output (MO) of the TMS stimulator (MagPro X100; MagVenture), corresponding to relative dI/dt (current rate‐of‐change) values of 75 × 10^6^ A/s. The default conductivity values were used (white matter: 0.126 S/m, gray matter: 0.275 S/m, cerebrospinal fluid: 1.654 S/m, scalp: 0.465 S/m, eyeballs: 0.5 S/m, compact bone: 0.008 S/m, spongy bone: 0.025 S/m, Blood: 0.6 S/m, and muscle: 0.465 S/m) (Puonti et al., 2020). The strength of the TMS‐induced EF was computed as the vector norm of the induced electric fields, which is believed to be the dominant factor for the activation of cortical neurons (Thielscher et al., 2011). We calculated an average EF strength in the ROIs as the measure: each ROI at the subject space was transformed from its corresponding MNI coordinate, and we subsequently extracted the average E‐field in a 5‐mm radius sphere in the gray matter in each model. Furthermore, we computed the ratio of the EF at each ROI during stimulation of the ROI relative to that at M1 during M1 stimulation. This allowed us to obtain a comparable stimulation intensity of each ROI in relation to M1 stimulation. Additionally, we analyzed the scalp‐to‐cortex (SCD) distance across each ROI, in order to investigate the impact of SCD on the strength of TMS‐induced EF. The SCD for each ROI was extracted from the SimNIBS output file.

**Figure 2:**
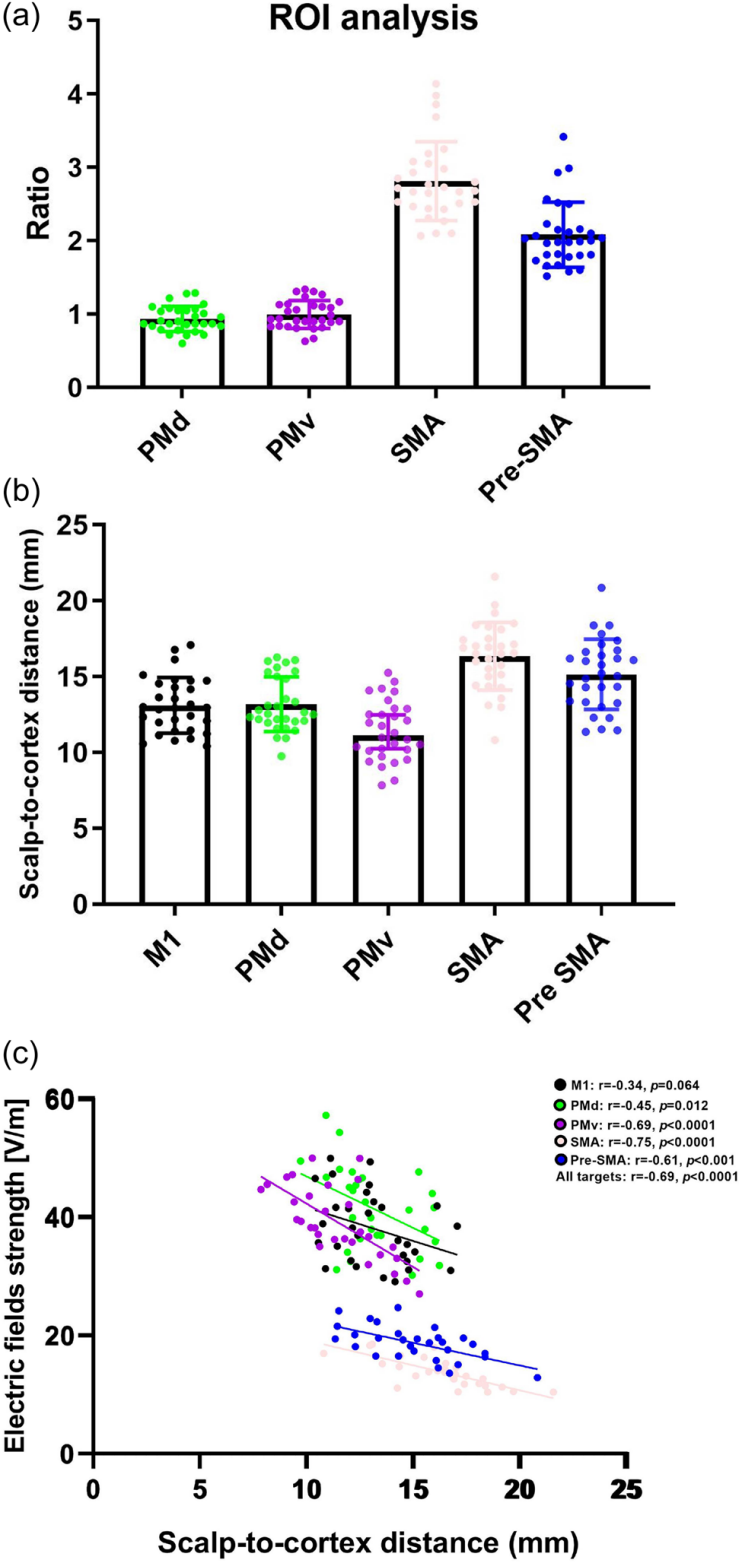
(a) The ratio of the equivalent EF at each ROI during stimulation to that at the M1 during M1 stimulation using ROI analysis. (b) The scalp‐to‐cortex distance across the five ROIs. (c) Correlation between SCD and TMS‐induced EFs in the five regions of interest. Each point is associated with a single subject. EF, electric field; M1, primary motor cortex; ROI, region of interest; SCD, scalp‐to‐cortex distance; TMS, transcranial magnetic stimulation.

Using ROI analysis, we found that an average of 94% RMT (range: 60%–129%) and 99% RMT (range: 63%–134%) was required for an equivalent stimulation intensity over the PMd and PMv, respectively, with reference to 100% RMT stimulation over the M1. An average of 281% RMT (range: 207%–414%) and 209% RMT (range: 152%–342%) were required for an equivalent stimulation intensity over the SMA and pre‐SMA, respectively, with reference to 100% RMT stimulation over the M1 (Figure 2a). F‐tests for equality of two variances revealed significant differences in variances among the following pairs: PMd versus SMA (*F* = 0.103, *p* < 0.001), PMd versus pre‐SMA (*F* = 0.125, *p* < 0.001), PMv versus SMA (*F* = 0.151, *p* < 0.001) and PMv versus pre‐SMA (*F* = 0.184, *p* < 0.001). SMA and pre‐SMA demonstrated larger SD values than PMd and PMv (SD values for PMd, PMv, SMA, and pre‐SMA were 0.172, 0.190, 0.537, and 0.443, respectively).

Given the high stimulation current providing equivalent exposure during stimulation over the SMA and pre‐SMA, we further conducted an investigation into the influence of SCD on the TMS‐induced EF over each ROI. Using paired *t* tests, we observed a significant difference in SCD between each pair, except for the comparison between SCD over the M1 and that over the PMd. The analyses of SCD distances across each ROI revealed a significant increase in SCD distance over the SMA and pre‐SMA compared to other cortical motor regions (Figure 2b). Using Pearson's correlation analyses, we consistently observed significant negative correlations between SCD and the strength of TMS‐induced EF over the secondary motor cortex (PMd, PMv, SMA, and pre‐SMA). The correlation between SCD and the strength of TMS‐induced EF over the M1 was marginally significant (Figure 2c).

The current study has a few limitations that should be acknowledged. On the one hand, the simulation was based on an MRI dataset from a non‐TMS experiment. Thus, we did not have individual RMT information. As a result, it is important to further validate our findings using data from actual TMS experiments. However, it is worth noting that we calculated the ratio within each subject, considering that the TMS‐induced EFs are linearly scaled to the current intensity during computational simulation. These results are expected to align with real‐world observations. On the other hand, the current data were obtained from a specific group of young healthy adults. Therefore, the extent to which the current findings can be generalized to older adults and populations with diseases remains uncertain.

In summary, the current computational simulation study indicates that the stimulation threshold of the premotor cortex, specifically the PMv and PMd, is close to that of the M1, with an averaged threshold of around 90% RMT; however, the exact threshold value varies among individuals. In addition, an intensity above 100% RMT would be required for effective SMA stimulation in a healthy brain. However, at the ROI level, the SMA stimulation resulted in a much lower EF. SCD was found to be a predominant factor influencing the strength of TMS‐induced EF over cortical motor areas. Due to the deeper location of the anatomical structures involved in SMA stimulation (as indicated by the significantly higher SCD), the use of a double‐cone coil is deemed more practical (Lu & Ueno, 2017). The great interindividual variation also indicates that simply utilizing a fixed intensity may lead to high response variability and poor treatment responses, which highlights the need of using simulated EF modeling to guide the decision‐making of TMS intervention for motor stimulation in neurological rehabilitation. Notably, the current study used computational modeling, and the results need to be examined with real‐world clinical data with individualized RMT.

## 1 CONCLUSION

We demonstrated a strong individual variability of simulated EFs induced by TMS targeting different cortical motor regions. Targeting SMA and pre‐SMA would require higher intensity stimulation due to increased SCD compared to the M1. Our simulation results underscore the risk of poor modulatory and therapeutic responses when using fixed intensity. Precise intensity adjustment, personalized based on subject‐specific EF modeling, is useful for optimizing TMS interventions targeting the secondary motor cortex to enhance cognitive‐motor functions in neurorehabilitation.

## AUTHOR CONTRIBUTIONS

**Jack Jiaqi Zhang**: Conceptualization; data curation; data analysis; visualization; writing—original draft. **Bella Bingbing Zhang**: Conceptualization; writing—review. **Zhongfei Bai**: Conceptualization; writing—review. **Kenneth N. K. Fong**: Conceptualization; writing—review.

## CONFLICT OF INTEREST STATEMENT

The authors declare no conflict of interest.

## ETHICS STATEMENT

Ethical approval is not applicable to the current computational simulation study. The original MRI study was approved by the Research Ethics Committee of the Institute of Applied Psychology at Jagiellonian University and Bioethics Commission of the Polish Military Institute of Aviation Medicine.

## Data Availability

The data that support the findings of this study are available on request from the corresponding author.
